# Identification of Small-Molecule Inhibitors against *Meso*-2, 6-Diaminopimelate Dehydrogenase from *Porphyromonas gingivalis*

**DOI:** 10.1371/journal.pone.0141126

**Published:** 2015-11-06

**Authors:** Victoria N. Stone, Hardik I. Parikh, Fadi El-rami, Xiuchun Ge, Weihau Chen, Yan Zhang, Glen E. Kellogg, Ping Xu

**Affiliations:** 1 Philips Institute for Oral Health Research, Virginia Commonwealth University, Richmond, Virginia, United States of America; 2 Department of Microbiology and Immunology, Virginia Commonwealth University, Richmond, Virginia, United States of America; 3 Department of Medicinal Chemistry, Virginia Commonwealth University, Richmond, Virginia, United States of America; 4 Center for the Study of Biological Complexity, Virginia Commonwealth University, Richmond, Virginia, United States of America; Oregon Health and Science University, UNITED STATES

## Abstract

Species-specific antimicrobial therapy has the potential to combat the increasing threat of antibiotic resistance and alteration of the human microbiome. We therefore set out to demonstrate the beginning of a pathogen-selective drug discovery method using the periodontal pathogen *Porphyromonas gingivalis* as a model. Through our knowledge of metabolic networks and essential genes we identified a “druggable” essential target, *meso*-diaminopimelate dehydrogenase, which is found in a limited number of species. We adopted a high-throughput virtual screen method on the ZINC chemical library to select a group of potential small-molecule inhibitors. *Meso*-diaminopimelate dehydrogenase from *P*. *gingivalis* was first expressed and purified in *Escherichia coli* then characterized for enzymatic inhibitor screening studies. Several inhibitors with similar structural scaffolds containing a sulfonamide core and aromatic substituents showed dose-dependent inhibition. These compounds were further assayed showing reasonable whole-cell activity and the inhibition mechanism was determined. We conclude that the establishment of this target and screening strategy provides a model for the future development of new antimicrobials.

## Introduction

Antibiotic resistance has become a prominent public health concern as it has reduced the effectiveness of current antimicrobials and led to increased mortality rates for previous treatable bacterial infections, e.g. multi-drug resistant tuberculosis [1]. Despite this fact, antimicrobial drug research has declined. On average, it requires approximately $800 million plus 10 or more years to bring a drug to market [2, 3]. Coupled with difficulties in target identification and drug screening methods, the pharmaceutical industry’s interest in antibiotic development has waned [3]. Meanwhile, we are beginning to understand the dynamics between the human microbiome and antibiotics more comprehensively. Microbes that makes up the human microbiome outnumber our cells by a factor of 10 to 1 [4] and studies show that they play critical roles in development [5] and maintaining human health [6]. Accordingly, the microbiome can be considered an essential part of our ecosystem that warrants consideration in dietary, genetic and medicinal elements. However, issues arise when there is a disruption in the homeostasis of the environment such as with the use of broad-spectrum antibiotics. Antibiotic therapy can affect both pathogenic and non-pathogenic species which disrupts the normal microbial population, resulting in various opportunistic infections, systemic co-morbidities and selects for bacterial resistance populations [7, 8]. Recent studies have shown that antibiotics taken at a young age can alter the gut microbiota, reducing the dominant species [9] and this change in species diversity can be long lasting, contributing to adverse effects like weight gain and the development of autoimmune disorders [10, 11].

New approaches in drug development research are critical to the future of antibiotics. Novel drugs that selectively target pathogenic species would offer an alternative to currently overused broad-spectrum antimicrobials. If an etiological agent can be identified within a poly-microbial environment, antimicrobials targeting a limited range of species not only will reduce the chances of resistance but also be more cost effective, reduce toxicity and allow for the maintenance of the healthy flora [12]. Advances in genomics, structural biology and computational chemistry have provided many novel approaches to target discovery and drug development [13]. Metabolic understanding of essential gene functions allow for the rapid prediction of essential genes as potential antimicrobial targets in a variety of organisms, even if experimental data is lacking [14]. This understanding coupled with knowledge of alternative pathways and differing metabolic requirements can be used to identify unique or species limited genes. Computer-based molecular modeling and structure-based virtual screening have become essential drug discovery tools that are part of successful rational drug design strategies in both industry and academia. When complemented with effective biochemical screening assays for binding and function, structure-based virtual screening can be a rapid, efficient and inexpensive way to identify and obtain a selection of potential inhibitors.

The oral cavity is one of the most diversified sites of the microbiome, consisting of 700–1000 phylotypes. Disruption in the microbial homeostasis leads to oral diseases such as periodontitis, a chronic inflammatory process. Periodontitis is characterized by the destruction of tooth supporting structures, bone resorption and the loss of tooth attachment [15]. It affects approximately 46% of the US adult population, 10% globally [16, 17] and is associated with systemic comorbidities such as pregnancy complications, arthritis, respiratory, cardiovascular and cerebrovascular diseases [18, 19]. Studies have shown that *Porphyromonas gingivalis*, a Gram-negative anaerobe, is a key pathogen in the development of this disease [20–22]. Therefore, we aimed to selectively target this organism within the oral cavity. Here we present an exploratory model for pathogenic-specific drug discovery using *P*. *gingivalis* and periodontal disease. We applied our knowledge of essential genes to predict a target limited to specific species and adopted a high-throughput virtual screening strategy utilizing the ZINC drug-like database of commercially available chemicals to identify small-molecule inhibitors. We then experimentally assessed the properties of the target and potential candidate inhibitors as the initial steps of developing a novel therapeutic approach.

## Materials and Methods

### Bacterial strains, plasmids and growth conditions

*P*. *gingivalis* W83 strain was cultured anaerobically (10% CO_2_, 10% H_2_, and 80% N_2_) at 37°C in tryptic soy broth (TSB) (Becton Dickinson, Franklin Lakes, NJ) supplemented with 1 μg/mL menadione and 5 μg/mL hemin. When appropriate, erythromycin (Fisher Scientific, Fair Lawn, NJ) was used at a concentration of 5 μg/ml. Plasmid pVA2198 (Richmond, VA) [23] was used to isolate the erythromycin resistance cassette.

### Primer design and recombinant PCR product construction

Primer design was based on the method by Xu *et al*. [14]. Two set of primers, F1/F2 (5’- CTC CGA ATA GCA AAC ATC TAC TG -3’ and 5’- GAA AAA TTT CAT CCT TCG TAG TCG AGC AGC CAT GCG C -3’) and F3/R3 (5’- GGG CAA TTT CTT TTT TGT CAT TTG TCA AAT CTG GGG G -3’ and 5’- GAT AAT CAT GCT TCG GAG ATG -3’), were designed to amplify a 1.2kb sequence upstream and downstream, respectively, of the *P*. *gingivalis* target gene. A third primer set, F2/R2 (5’- GCG CAT GGC TGC TCG ACT ACG AAG GAT GAA ATT TTT C -3’ and 5’- CCC CCA GAT TTG ACA AAT GAC AAA AAA GAA ATT GCC C -3’) was designed to amplify the 800 bp erythromycin resistance cassette (Erm^R^) containing the *ermF* gene from plasmid pVA2198. To minimize polar effects from mutagenesis, primers were designed to include stop codons within frame and the antibiotic resistance cassette was designed to run in the same orientation as the target gene to ensure transcription. The three PCR fragments were created using F1/R1, F2/R2 and F3/R3. All PCR reactions were performed with an initial denature of 98°C for 30 sec, 30 cycles of 98°C for 10 sec, 56°C for 30 sec, 72°C for 36 sec and a final extension of 72°C for 7 min. The PCR products were purified using QIAquick PCR Purification Kit (Qiagen, Valencia, CA); the three fragments were then combined in equal amounts and amplified again using F1 and R3 primers to generate the final linear recombinant product. The PCR reaction was performed with an initial denature of 98°C for 30 sec, 30 cycles of 98°C for 10 sec, 56°C for 30 sec, 72°C for 1 min 36 sec and a final extension of 72°C for 7 min. Phusion High-Fidelity Taq DNA polymerase (New England Biolabs, Ipswich, MA) was used in all reactions.

### Transformation

The electroporation method was adapted from Fletcher *et al*. [23]. Briefly, 0.2 ml of an actively growing culture of *P*. *gingivalis* was used to inoculate 2 ml of BHI broth supplemented with yeast extract, hemin and menadione, which was then incubated overnight at 37°C. Five milliliters of the same medium pre-warmed at 37°C was then inoculated with 0.5 ml of the overnight culture and was incubated for an additional 4 h (OD_600_ ≈0.7). The cells were harvested by centrifugation at 6,000 x *g* for 15 min at 4°C and washed twice in 10 ml of ice-cold electroporation buffer (10% glycerol, 1 mM MgCl_2_). The final cell pellet was re-suspended in 0.5 ml of electroporation buffer. A 100 μl sample of re-suspended cells and 5 μg of DNA were placed in a sterile electrode cuvette (0.2-cm gap). The cells were then pulsed with a Bio-Rad (Hercules, CA) gene pulser at 2,500 V for 9.5 ms and incubated on ice for 5 min. The cell suspension was then added to 0.6 ml of BHI broth supplemented with yeast extract, hemin and menadione and incubated for approximately 16 h. A 100 μl sample was plated on agar medium containing erythromycin and was incubated anaerobically at 37°C for 5 to 10 days.

### Multiple sequence alignment

For the prediction of the substrate binding site, the protein database, UniProtKB/Swiss-Prot (www.uniprot.org) [24], was referenced for organisms with completed enzymatic and functional data for *m*-Ddh. This included *Corynebacterium glutamicum* ATCC 27405, *Lysinibacillus sphaericus*, *Bacteroides fragilis* ATCC 25285, *Clostridium thermocellum* ATCC 13032 and *Ureibacillus thermosphaericus* (Uniprot ID: P04964, Q9KWR0, Q5L9Q6, A3DDX7, G1UII1). Complete protein sequences were obtained from the National Center for Biotechnology Information (NCBI, www.ncbi.nlm.nih.gov/) database. The multiple sequence alignment analyses were then performed using the T-Coffee multiple alignment [25]. The alignment figure was generated in ESpript 3.0 [26] for visualization.

### Cloning, expression and purification of *m*-Ddh

The amino acid sequence of *m*-Ddh from *P*. *gingivalis* was codon-optimized for expression in *E*. *coli* cells, synthesized and cloned into a pUC57 vector by GenScript (Piscataway, NJ). To introduce the 6×-HIS tag to the C-terminal end of the gene, the plasmid was digested at *NdeI* and *NotI* restriction sites. The digested fragments were loaded onto a 1% agarose gel and purified using MinElute® Gel Extraction kit (Qiagen, Valencia, CA). The purified DNA insert was ligated into a *NdeI-* and *NotI*-digested pET-21a (+) vector (Merck Millipore, Billerica, MA) by T4 DNA ligase (New England Biolabs, Ipswich, MA), yielding the expression plasmid pET-Ddh. The plasmids containing the DNA construct were isolated using QIAprep® Spin Miniprep plasmid (Qiagen, Valencia, CA) and sequenced at VCU Nucleic Acids Research Facilities (Richmond, VA).

The pET-Ddh plasmid was introduced into *E*. *coli* BL21 (DE3) pLysS (BioLine, Taunton, MA) and grown overnight in auto-inducing media ZYP5052 containing 100 μg/ml ampicillin at 37°C. For purification, cells were disrupted by Emulsiflex C3 high pressure emulsifier (Avestin, Ottawa, Canada). Soluble protein was collected and separated from cell debris by centrifugation (20, 000 × *g* for 20 mins at 4°C). The resulting supernatant was loaded onto a NTA-Ni^2+^ affinity column (Qiagen) pre-equilibrated with running buffer (25 mM Tris, 300 mM NaCl, 10 mM imidazole, pH 8.0). Unbound protein was washed off with wash buffer (25 mM Tris, 300 mM NaCl, 10 mM imidazole, pH 8.0) and chelated protein was eluted off with elution buffer (25 mM Tris, 300 mM NaCl, 100 mM imidazole, pH 8.0). Protein concentration was calculated based off of absorbance at 280 nm. Purified protein was boiled in 2X Laemmli buffer (4% SDS, 10% β-mercaptoethanol, 20% glycerol, 0.125 M Tris-HCl, 0.004% bromophenol blue) and visualized by 12.5% sodium dodecyl sulfate-polyacrylamide gel electrophoresis (SDS-PAGE) stained with Coomassie Blue G-250 (Bio-Rad, Hercules, CA).

### *m-*Ddh kinetic assay

The enzymatic activity for *m*-Ddh was determined by observing the standard oxidative deamination reaction of the substrate *meso*-diaminopimelate [27, 28]. The reaction contained 400 μM of *meso*-diaminopimelate (Sigma-Aldrich, St. Louis, MO), 180 μM NADP^+^ (Sigma-Aldrich, St. Louis, MO), 200 mM glycine-KCl-KOH buffer (pH 10.5), and the enzyme in a final volume of 1 ml. The reaction was initiated with the addition of NADP^+^. The reaction velocity was calculated from the increase in absorbance at 340 nm, spectrophotometrically monitored at 25°C, where one unit of enzyme was defined as the amount of enzyme catalyzing the formation of 1 mmol of NADPH per min.

### Determination of kinetic parameters

Initial velocity measurements for *m*-DAP and NADP^+^ were determined at 25°C in a similar reaction for the standard oxidative deamination reaction assay. The reaction contained 200 mM glycine-KCl-KOH buffer (pH 10.5) with *m*-DAP as the variable substrate with concentrations between 0.001 mM and 1 mM and NADP^+^ held constant at a saturating concentration of 0.5 mM or NADP^+^ as the variable substrate with concentrations between 0.01 mM to 1 mM and *m*-DAP held constant at 0.5 mM. K_m_ and V_max_ values were determined through non-linear fitting. All assays were performed in triplicates and non-linear fitting Michaelis-Menten data were calculated from Graphpad Prism version 5.04 (Graphpad, San Diego, CA).

### Molecular modeling

#### Protein structure

The structure of *meso*-diaminopimelate dehydrogenase (oxidoreductase, Gfo/Idh/MocA family member) from *P*. *gingivalis* strain W83, was crystallized as part of the National Institute of Health-National Institute of General Medical Sciences (NIH-NIGMS) sponsored Protein Structure Initiative (http://www.nigms.nih.gov/Initiatives/PSI/) [29, 30] and was solved at a resolution of 1.80 Å. The crystal structure data was downloaded from the Protein Data Bank (PDB ID: 3BIO) and applied in our study. The binding site was identified by sequence homology to the ortholog in *C*. *glutamicum*, whose crystal structure was previously determined in a complex with the substrate, *meso*-diaminopimelate (*m*-DAP), in the binding pocket (PDB ID: 2DAP) [31]. Using Sybyl X.1 (Tripos, St. Louis, MO), the protein was prepared for virtual screening and docking studies by extracting water molecules and co-crystallized ligands and deleting one of the two monomers. The pKa values of the amino acid residues within the binding pocket were predicted and the appropriate ionization states were assigned in SYBYL for a pH 10.5, determined based on the conditions of the *in vitro* experimental assay. Appropriate atom types were assigned, hydrogens were added and the protein was minimized with Sybyl’s Tripos force field.

#### Structure-based virtual screening

Virtual screening was performed with the UNITY module within the Sybyl-X molecular modeling program. Unity uses a directed tweak algorithm [32] to simulate molecular flexibility while screening small-molecules. The binding pocket of *m*-Ddh was used as the target site, by constructing a variety of queries based on the pocket’s properties. Over 9 million small-molecules were screened *in silico* from ZINC [33] drug-like databases (http://www.zinc.docking.org).

#### Molecular docking

Docking and two-step scoring was used to evaluate the results of virtual screening. By visually inspecting and filtering the UNITY hits, we selected the top 132 small-molecule inhibitors for further computational analysis. We used GOLD (Genetic Optimization Ligand Docking) docking program (Version 5.2) [34], targeting the binding site of in *m-* Ddh. A sphere with radius of 12 Å from Arg183 was set as the docking region. This allowed for the inclusion of all residues expected to be within the binding site. The protein model was prepared for docking as described above. Conformational flexibility was allowed for the small-molecules. We allowed for 50 GA (Genetic Algorithms) runs with a distance of 1 Å between clusters. The 132 compounds selected from our virtual screening hits were docked by GOLD, ranked by Goldscore and then re-ranked by the CHEMPLP as implemented in GOLD. All docked compounds were then scored in a second pass by HINT (Hydropathic INTeractions) [35]. The binding mode corresponding to the highest-ranking HINT score for each compound was chosen as the best and most likely conformation for that compound. From these 132 compounds, the top 30% of the best-scored, structurally diverse compounds as ranked by HINT were re-docked and minimized with 10,000 iterations within the *m*-Ddh binding site. Finally, out of forty top scored small-molecules, 11 compounds were commercially available and were purchased for assay. All modeling images were generated in Pymol (http://www.pymol.org).

### Compounds

The selected compounds were purchased from Vitas-M Laboratory, Ltd. (Moscow, Russia), Molport (Riga, Latvia) and/or eMolecules (La Jolla, CA, USA), which reported compound purities over 90%, analyzed by NMR and/or LC- MS. All compounds were re-suspended in DMSO (Sigma–Aldrich, St. Louis, MO, USA) prior to use.

### Inhibitor screening and determination of IC_50_ values

A range of concentrations (0–3 mM) of each small-molecule compound inhibitor were added to the standard reaction and the percent of *m*-Ddh enzymatic inhibition was measured by the kinetic assay described above. Percent inhibition was determined by the formula: $\frac{\left(\;{rate\;of\;reaction}_{no\;inhibitor}\;)-({rate\;of\;reaction}_{inhibitor}\right)}{{rate\;of\;reaction}_{no\;inhibitor}}\times \;100.$ The concentration of each compound inhibitor which caused 50% enzymatic inhibition (IC_50_) was calculated using PRISM v6.04 software (Graphpad, San Diego, CA) from three independent experiments.

### Determination of antimicrobial properties

Minimal Inhibitory Concentration (MIC) assays were performed using a broth microdilution method [36]. *P*. *gingivalis* cells were grown overnight and the following day diluted 1/10 into fresh medium. Cells were allowed to grow to mid-log phase (OD_600_ ≈ 0.5). Compounds were serially diluted in 96-well microtiter plates (Jet Biofil, Genesee Scientific, San Diego, CA) and an aliquot of the cell suspension was added to each well with the compound inhibitor sample for a final cell count of 1×10^5^ CFU/ml. Plates were incubated for five days at 37°C in anaerobic conditions. The MIC was defined as the lowest concentration of compound inhibitor that visually reduced cell growth relative to the controls.

Minimal bactericidal concentrations (MBC) were determined by plating bacteria from wells of the MIC assay that showed no visible growth. Samples were plated on tryptic soy agar plates supplemented with 5% sheep blood (Becton, Dickinson, Franklin Lakes, NJ) and incubated at 37°C in anaerobic conditions for 7 days. MBC was defined as the lowest concentration of compound inhibitor that resulted in no colony formation/growth.

### Time-kill assay

*P*. *gingivalis* cells were grown overnight and the following day diluted 1/10 into fresh medium. Cells were allowed to grow to mid-log phase (OD_600_ ≈ 0.5) then diluted to a final cell suspension of 1×10^5^ CFU/ml. Compounds were added at a concentration of 5x the MIC determined from the 96-well broth microdilution assay. Samples were taken at different time intervals (0, 0.25, 0.5, 1, 2, 3, 4, 6 and 24 hours) and plated on tryptic soy agar plates supplemented with 5% sheep blood (Becton, Dickinson, Franklin Lakes, NJ) using an automated Eddy Jet spiral plater (Neutec Group, Farmingdale, NY). Plates were incubated at 37°C in anaerobic conditions for 7 days.

### SEM analysis of compound exposed *P*. *gingivalis* cells

Untreated or treated *P*. *gingivalis* cells were deposited onto a 0.1 μm disposable Millipore filter to remove medium, and samples were fixed using 2% glutaraldehyde in 0.1 M sodium cacodylate buffer (pH 7.4) for 30 min, followed by 1% osmium tetroxide in 0.1 M sodium cacodylate buffer (pH 7.4). Samples embedded in the filters were then dehydrated in ethanol followed by hexamethyldisilazane (HMDS) and allowed to air-dry. The filters were sectioned and mounted onto stubs and coated with gold for three minutes (EMS– 550 Automated Sputter Coater, Electron Microscopy Sciences, Hatfield, PA). Micrographs were taken at 30,000× total magnification using a Zeiss EVO 50 XVP scanning electron microscope (Carl Zeiss, Peabody, MA).

### Analysis of inhibition mechanisms

Kinetic studies were carried out using the standard kinetic assay for the oxidative deamination of *m*-DAP. Reactions were performed in the absence or presence of compound inhibitors (0–0.4 mM) with varying concentrations of the substrate *m*-DAP or co-substrate NADP^+^. The mode of inhibition was determined from non-linear regression using PRISM v6.04 software (Graphpad, San Diego, CA) from three independent experiments. The mode of inhibition was graphically visualized with Lineweaver-Burk or Hanes-Woolf plots according to Cleland kinetics [37].

## Result

### Identification and assessment of *m*-Ddh as a target

Based on what we know of bacterial metabolism, the lysine biosynthesis pathway presented an attractive option for pathogen-selective targeting. Lysine is a required amino acid for bacteria and the pathway is directly involved in the biosynthesis of peptidoglycan. However, what makes lysine biosynthesis suitable for species selective targeting is the presence of pathway variants. The pathway is composed of four different branches, differing by the substrate intermediates at the branch point of L-2, 3, 4, 5-tetrahydrodipicolinate’s (THDP) conversion to *meso*-diaminopimelate (*m*-DAP). The succinylase branch utilizes succinyl-CoA to generate succinylated intermediates; similarly the acetylase branch utilizes acetyl-CoA to produce acetylated intermediates. These two variants are used by the majority of Gram-negative and Gram-positive bacteria. The aminotransferase branch, used by plants and methanococci, involves a single step amine transfer to produce the precursor of *m*-DAP, LL-DAP [38]. However, limited to a few species, *m*-DAP is directly produced by the enzyme *meso*-diaminopimelate dehydrogenase (*m*-Ddh; GenBank ID: AAQ65966.1) in a single step [10, 39–42]. As the healthy oral cavity is composed of roughly 80% streptococcus species, the absence of *m*-Ddh in streptococci while present in the key periodontal pathogen, *P*. *gingivalis*, indicated this enzyme could be a suitable target for periodontal disease.

**Fig 1:**
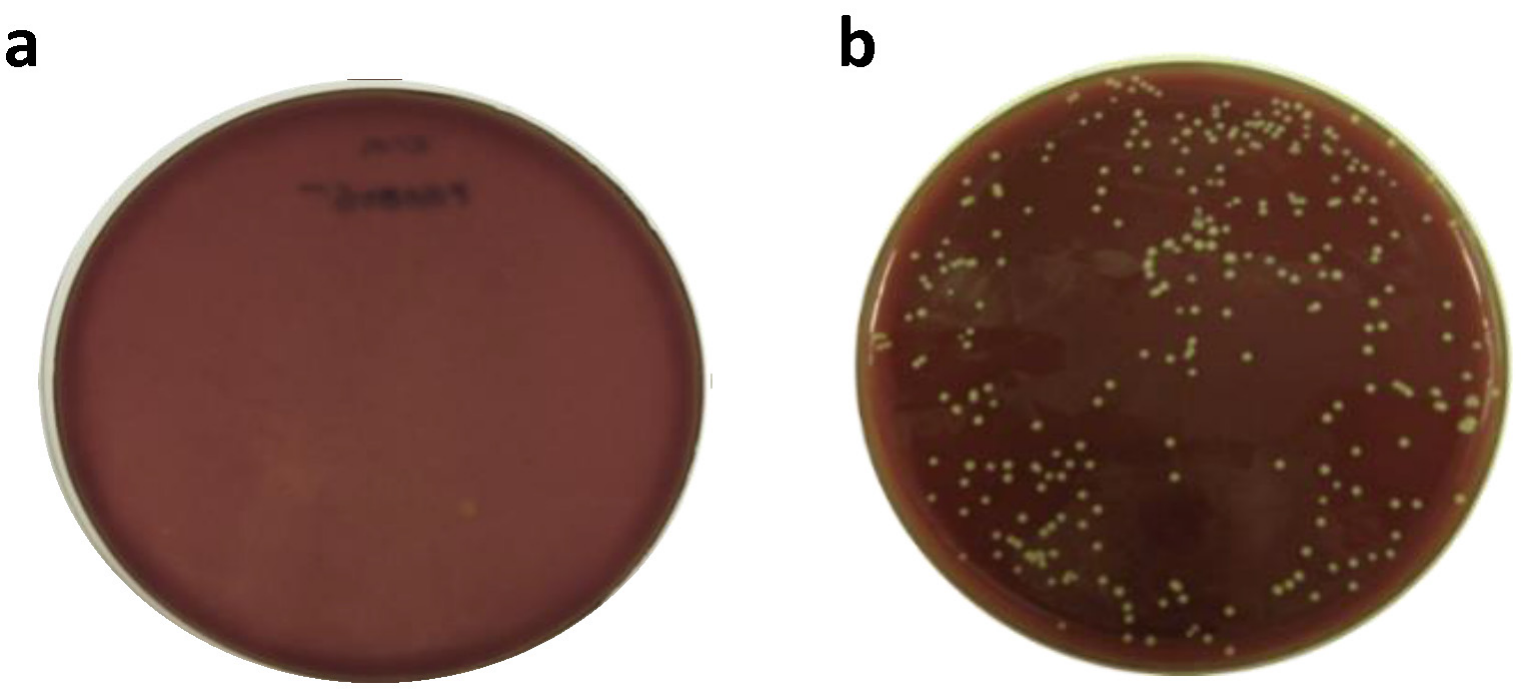
Allelic replacement mutagenesis for predicted essential and non-essential gene. (a) Predicted essential gene PG0806 was transformed and plated on antibiotic selective media resulting in no colony formation, validating prediction that the gene was essential. (b) Predicted non-essential gene PG0027 was transformed and plated on antibiotic selective media resulting in colony formation, validating prediction that the gene was not essential. Transformations were performed two independent times.

An important component in antimicrobial therapy is the ability to target critical biological processes required for bacterial cell survival. Historically, these targets have focused on key biological functions such as DNA replication, protein translation and cell wall biosynthesis [43]. While *m*-Ddh is involved in protein and cell wall biosynthesis and we predicted the gene to be essential, prior to the start of our study we did not know whether this was experimentally true. To verify *m*-Ddh was essential in *P*. *gingivalis* W83, we knocked out the gene by transforming cells with a recombinant PCR product carrying an erythromycin resistance cassette (Erm^R^) allowing for allelic replacement mutagenesis within the genome. Through this method, the antibiotic resistance cassette replaces the target gene and, if essential, results in non-viable cells following transformation. The disruption of the PG0806 gene with the Erm^R^ cassette resulted in no colony formation following electroporation and recovery in selective media (Fig 1A). To show that the lack of colony formation was in response to the essential nature of the gene and not due to problems with the transformation or electroporation, we simultaneously carried out allelic replacement mutagenesis for a non-essential hypothetical membrane protein (GenBank ID: AAQ65282.1). For this control we were able to obtain substantial colony formation (Fig 1B). These result were repeated independently displaying similar results as shown in Fig 1; suggesting that deletion of PG0806 is lethal to *P*. *gingivalis* and therefore essential.

**Fig 2:**
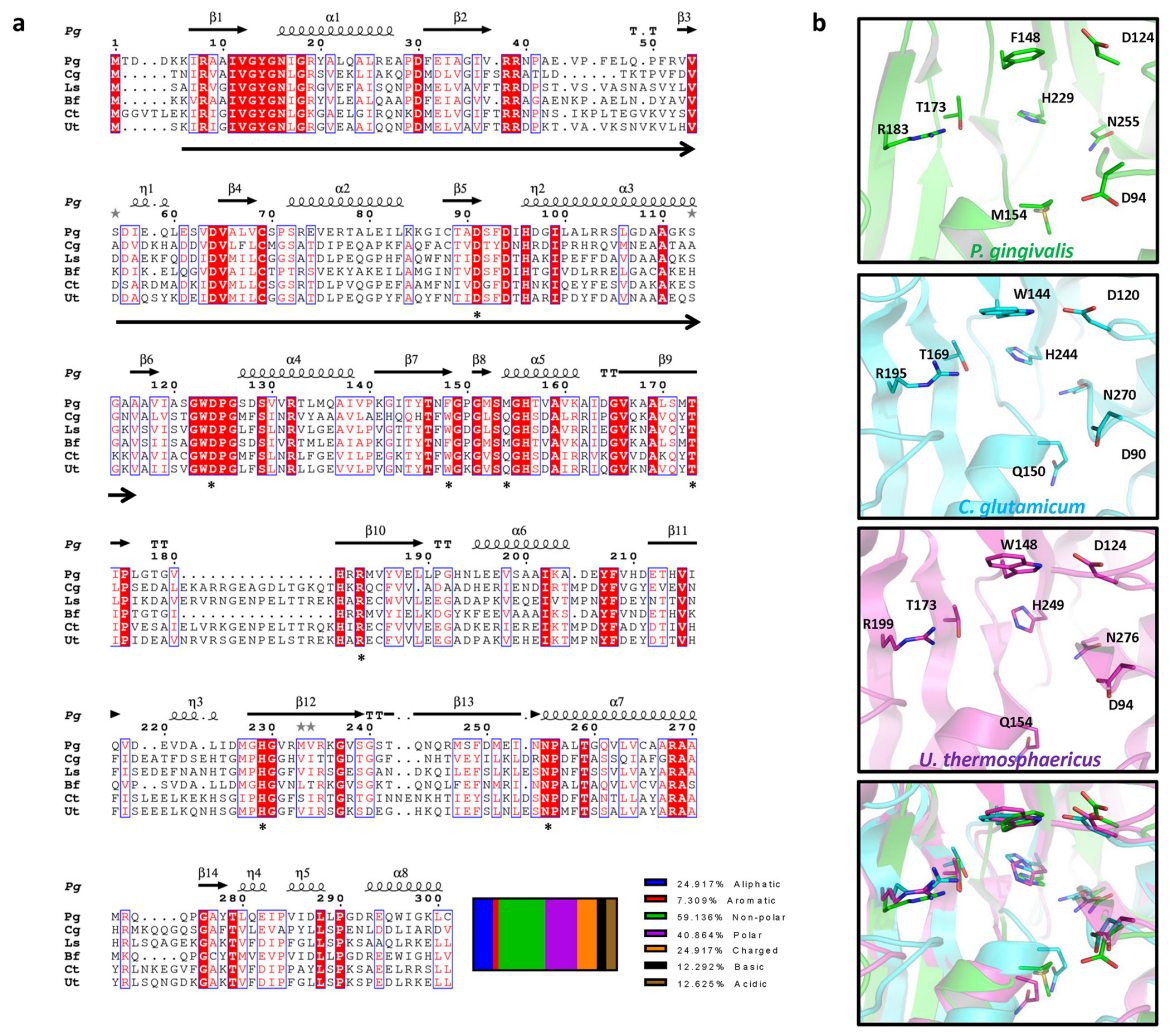
Protein sequence and structural alignment of *m*-Ddh. (a) Sequence alignment of *m*-Ddh from other bacterial organisms. Sequences were aligned using T-Coffee and the multiple alignment was then created in Espript 3.0. The putative binding sites of *Corynebacterium glutamicum* (Cg), *Lysinibacillus sphaericus* (Ls), *Bacteroides fragilis* (Bf), *Clostridium thermocellum* (Ct) and *Ureibacillus thermosphaericus* (Ut) cited in the sequence annotations in UniProtKB/Swiss-Prot and *P*. *gingivalis* (Pg) predicted based on homology are indicated by astericks. The oxidoreductase domain for *P*. *gingivalis* is indicated by arrows below sequence. Secondary structure for P. gingivalis is annotated above the sequence. Relative percentage of characterized amino acid residues are shown below. (b) Secondary structure alignment of *m*-Ddh’s putative binding site from *P*. *gingivalis* (green), *C*. *glutamicum* (cyan) and *U*. *thermosphaericus* (purple). Key residues are labeled, side chains are displayed as sticks and colored corresponding to atom type. Hydrogens were omitted for clarity.

**Fig 3:**
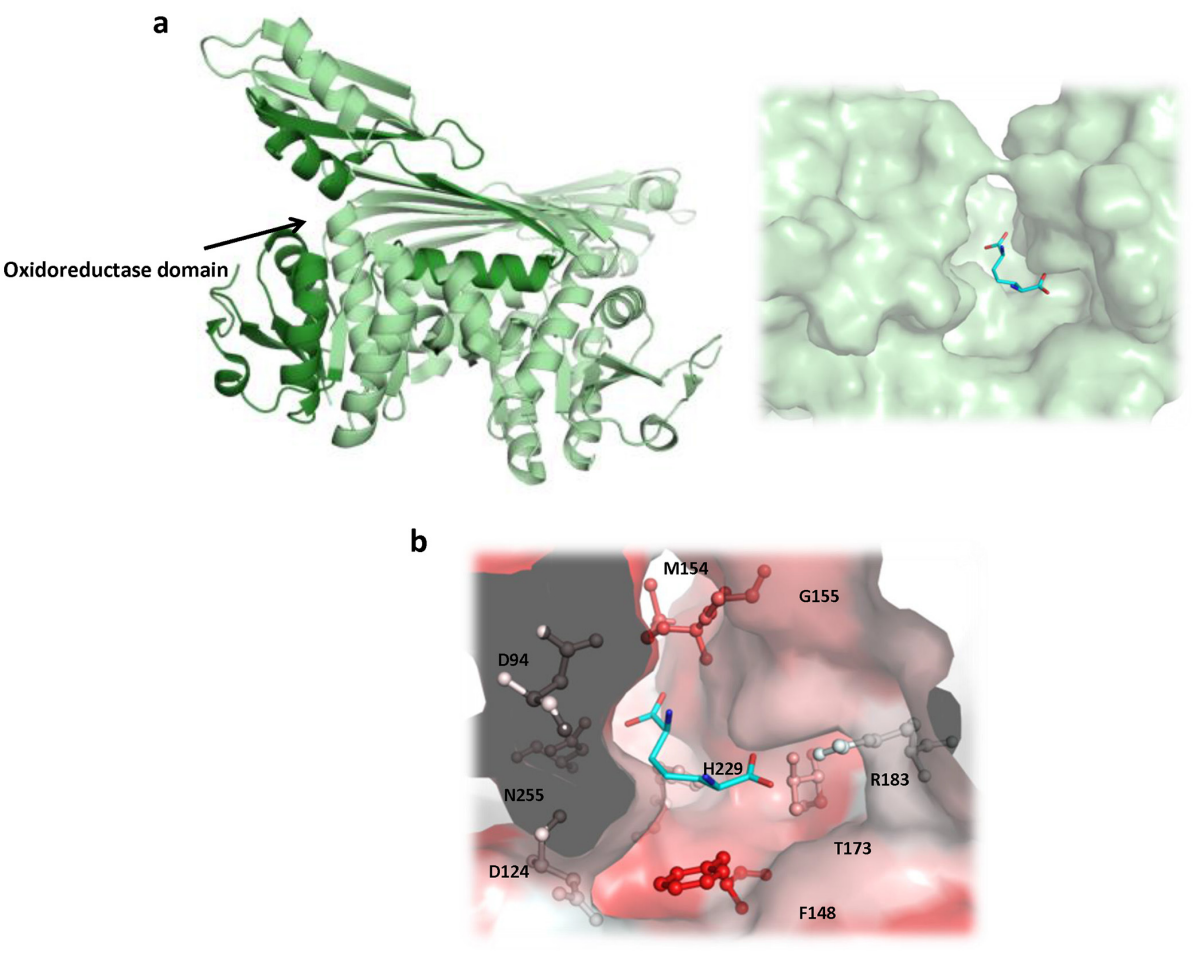
Cartoon representation of *m*-Ddh protein structure. (a) Ribbon based structure of *m*-Ddh with a zoomed in view of the substrate *m*-DAP binding cavity (b) and the hydrophobicity of binding site protein surface (red = hydrophobic).

Another important component for a potential target is “druggability” corresponding to the chance a small molecule will be able to bind and have a significant effect on the protein’s activity [44]. Druggability can be assessed in several methods including previous proof-of-concept in similar proteins, conserved or targetable sequence motifs and structural analysis [45]. *m*-Ddh enzymes from several organisms referenced in UniprotKB/Swiss-Prot with known or binding sites predicted by similarity were aligned (Fig 2A). *P*. *gingivalis* has approximately 30% sequence identity to *C*. *glutamicum*, *L*. *sphaericus*, *C*. *thermocellum* and *U*. *thermosphaericus* and 70% sequence identity to *B*. *fragilis*. Alignment of the secondary structure revealed that the binding site and residues are maintained across species, indicating that the enzyme and its function is highly conserved (Fig 2B). Druggable proteins have been shown to consist of a higher ratio of non-polar to polar amino acid residues (59.1% vs 40.9%) and a lower isoelectric point (5.39 vs 7.44 in non-targets) [46]. Analysis showed that *m*-Ddh contains an oxidoreductase domain, a favored targeted enzyme class [46] and which has been previously determine with high confidence to be “druggable” by the Druggable Cavity Directory. Structural analysis by SYBYL revealed a solvent inaccessible binding cavity with residues corresponding to the *m*-DAP sequence alignment and the conserved motifs (Fig 3A). This binding cavity consists of a relatively deep and hydrophobic region consisting of hydrophobic amino acid residues methionine, tryptophan and phenylalanine (Fig 3B). In addition, *m*-Ddh from *C*. *glutamicum* has been co-crystallized in a complex with the endogenous substrate [31]. Therefore, *m*-Ddh structure appears to be “druggable”, making it a suitable target for drug discovery.

As the role of an antimicrobial compound is to alter the functional behavior of its target, understanding the biological function of the enzyme plays a critical role in the drug design and discovery process. Therefore, in order to monitor changes in behavior and establish an assay for target-based screening to support the enzyme’s role as a potential antimicrobial target, we needed to first purify and characterized the protein and its enzymatic activity. To obtain a large amount of recombinant *P*. *gingivalis m*-Ddh protein for characterization, we synthesized the gene and expressed it in *E*. *coli*. The gene sequence encoding for *P*. *gingivalis m*-Ddh was codon-optimized and cloned into a T7 pET-21a (+) expression vector carrying a C-terminal 6× HIS tag. The protein was isolated following the expression and purification described in Materials and Methods. Expression from *E*. *coli* BL21 (DE3) pLysS cells at 37°C overnight resulted in the majority of the protein found within the soluble fraction. A 2-L culture produced approximately 16 mg of protein, determined by spectrophotometric analysis. The protein sequence of *m*-Ddh is 301 amino acid residues with a calculated molecular weight of 32 kDa, corresponding to the SDS-PAGE analysis of the purified protein (S1 Fig).

*m*-Ddh catalyzes the reversible NADP^+^-dependent oxidative deamination of the D-amino acid center of *meso*-diaminopimelate to produce L-α-amino-ε-ketopimelate and NADPH [27]. The production of NADPH allows for the continuous spectrophotometric monitoring of the enzymatic activity by measuring the change in absorbance at 340 nm as NADP^+^ is converted to NADPH. We examined the steady state kinetics to determine the kinetic parameters of the reaction. Analysis of the initial velocity showed typical Michaelis-Menten kinetics (S2 Fig). The apparent K_m_ and V_max_ for *m*-DAP was determined to be 370 μM and 130.1 nmol·sec^-1^ and 60 μM and 91.95 nmol·sec^-1^ for NADP^+^ respectively.

### Pharmacophore model and virtual screening for the identification of small-molecule compound inhibitors

To identify a binding model for inhibition, we searched the literature for known inhibitors. Unsaturated analogues of *m*-DAP, containing a planar α-carbon and lacking the active D-amino acid amine center of *m*-DAP have been shown to be strong inhibitors of *m*-Ddh isolated from *Bacillus sphaericus* and *C*. *glutamicum* [47]. It was assumed the analogue inhibitors bind in manner opposite to that of the substrate; thus, the non-reactive L-amino acid center is positioned near the C-4 position of the co-substrate NADP^+^. This would prevent the oxidation reaction and hydride exchange that normally would occur between the substrate and co-substrate. We obtained two of these previously reported compounds; testing *in vitro* showed dose-dependent inhibition against *m*-DAP from *P*. *gingivalis* (S3 Fig).

**Fig 4:**
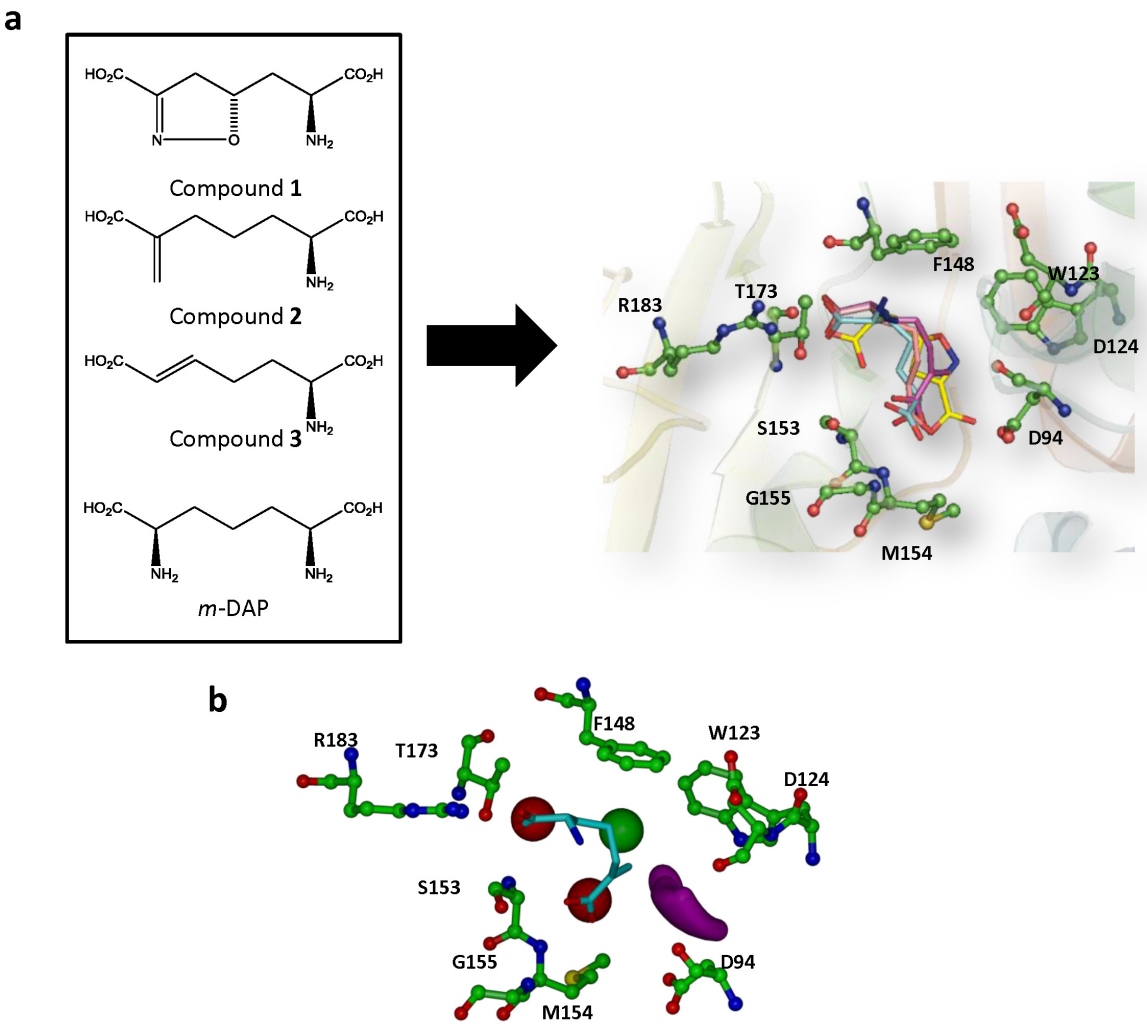
Generation of pharmacophore model for the high-throughput virtual screen. (a) Structure of *m*-DAP and inhibitor analogs that were previously shown to be active against *m*-Ddh in *C*. *glutamicum* and *B*. *sphaericus*. (b) Compounds docked into *m*-Ddh binding site and conserved interactions were identified. (c) Pharmacophore model with selected core features for inhibitor identification during virtual screen. The model focused on four features: first, a hydrophobic region complementary to amino acid residues Trp123 and Phe148 (green), second, a ligand donor atom complementary to residues Asp94 and Asp124 (purple), third, a negative center complementary to the side chain of residue Ser153 and the backbone of residues Met154 and Gly155 (red) and fourth, a negative center complementary to the side chain of residues Arg183 and Thr173 (red). The interaction was also restricted for an area 12Å in distance for Arg183. Key residues are labeled, displayed as ball and sticks and colored corresponding to atom type. Hydrogens were omitted for clarity.

The X-ray crystal structure was modeled and the unsaturated analogue inhibitors (Compounds **1**–**3**) as well as the *m*-DAP substrate were docked into the *m*-Ddh binding pocket to identify the features that should be important for inhibitor interactions (Fig 4A). The docking model which best fit the expected *in vitro* interaction and displayed high docking scores was used to generate a pharmacophore model. Based on the best ranking interaction, the pharmacophore model focused on four features that were shared between the inhibitors and substrate: 1) a hydrophobic region complementary to amino acid residues Trp123 and Phe148; 2) a ligand donor atom complementary to residues Asp94 and Asp124; 3) a negative (acceptor) center complementary to the side chain of residue Ser153 and the backbone of residues Met154 and Gly155; and 4) a negative (acceptor) center complementary to the side chains of residues Arg183 and Thr173. The interaction was also restricted to a sphere of radius 12 Å centered around Arg183. Interactions with Arg183 were assumed to be critical for the interaction because preliminary site-directed mutagenesis decreased the substrate-protein binding affinity by 32-fold [Stone *et al*., unpublished]. From our docking studies, Arg183 was seen to form hydrogen bonds with the carboxylate groups of the substrate analogues.

The pharmacophore model shown in Fig 4B was used in a high-throughput virtual screen of the ZINC 3D database [33] to identify small-molecule inhibitors that would fit the query constructed from the pharmacophore. ZINC (Zinc Is Not Commercial) is a publicly available listing of molecules that are reportedly available for purchase, organized in a manner appropriate for virtual screening studies. In simple terms, a compound is classified as a hit if it fits all of the features defined as mandatory in the model. The screening of more than 9 million compounds within the ZINC database resulted in more than several hundred hits.

**Fig 5:**
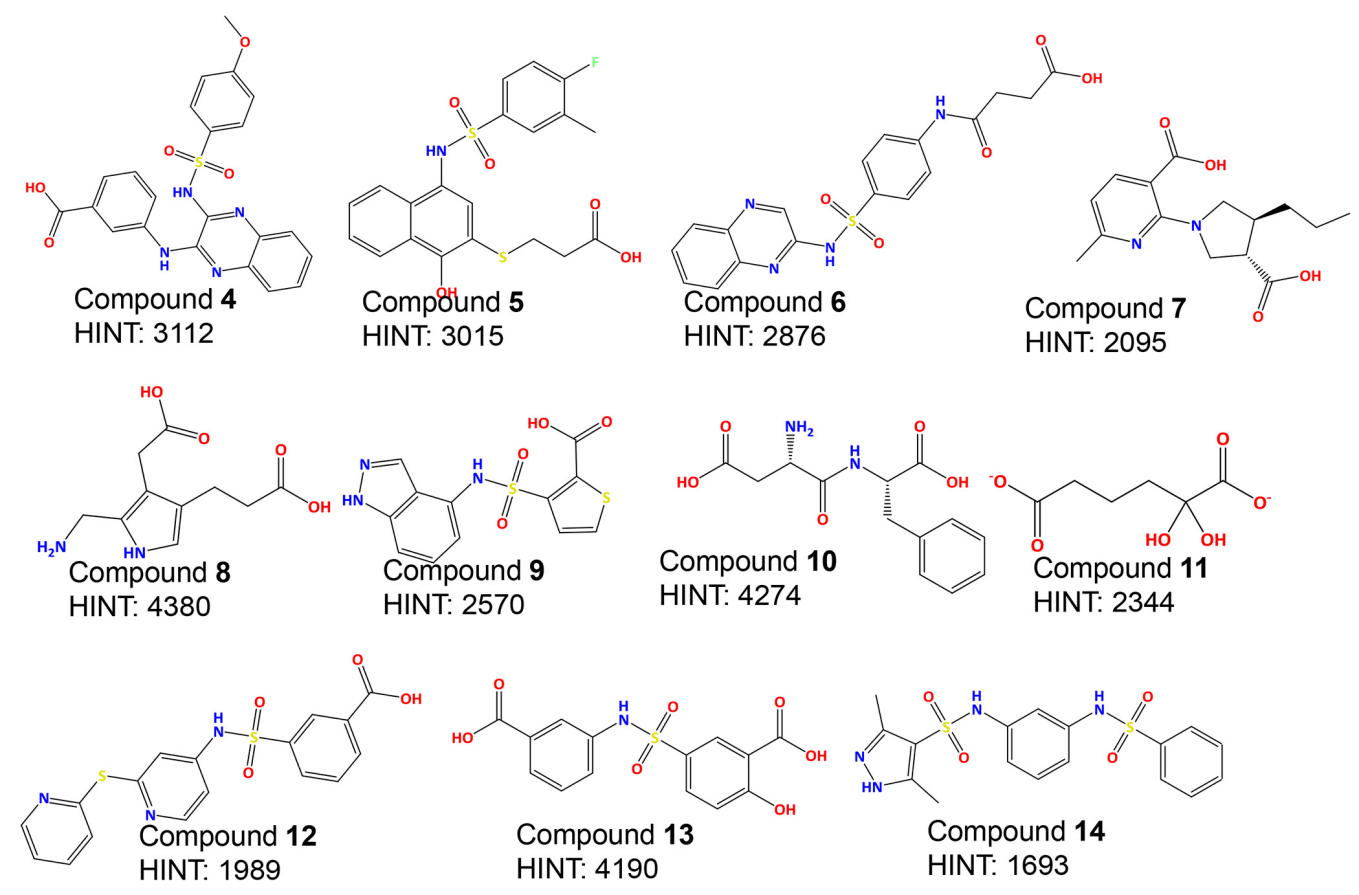
Structure and scoring of top-ranking inhibitors.

Since the goal of virtual screening is to identify unique compounds and scaffolds that have the potential to be developed into active inhibitors, a filter was applied to remove compounds within the hit list too structurally similar to one another. The resulting list was then filtered for drug likeness (i.e., with algorithms based on Lipinski’s Rule of Five [45]) to remove compounds and scaffolds that were unlikely to have reasonable physiochemical properties. Compounds passing through these filters were then docked to the binding site of *m*-Ddh with GOLD (Genetic Optimization of Ligand Docking) program [34] to predict their binding affinities and to assess the modeled compound-protein interactions. Prediction of the best fit binding model of each compound, again within 12 Å of Arg183, was determined and scored by Goldscore. The models with top docking scores were re-docked to the binding site with the same docking parameters and rescored by CHEMPLP. A filter based on binding pose was applied and molecules that interacted favorably, mostly via hydrogen bonds with the key residue Arg183 were identified. This filter yielded 132 hits, which were then scored by the HINT (Hydropathic INTeractions) force field [35]. HINT uses an empirical force field that estimates the free energy of intermolecular interactions based on small molecule partition coefficients (Log P_o/w_). The program accounts for the sum all non-covalent interactions, quantitatively evaluating both favorable and unfavorable interactions. Therefore, the higher the score the more favorable the overall interaction. HINT has been used for over 20 years as a scoring tool and has shown to reliably correlate with the free energy of docked molecules and binding efficiency [35, 48, 49]. The binding mode corresponding to the highest HINT score for each compound was then re-docked and minimized within the *m*-Ddh binding site. From these 132 compounds, the top 30% of the best HINT-scored, structurally diverse compounds were set-aside as the 48 final hits. Finally, samples of the commercially available compounds in this group were purchased for screening assays as described below. The HINT scores and compound structures of each of these 11 compounds (**4**–**14**) are listed in Fig 5.

### Evaluation of compound enzymatic and cellular inhibition against *m*-Ddh

The initial screens for the compounds **4**–**14** were performed by individually adding each compound to the assay solution at a concentration of 3 mM. Enzymatic activity was measured by the standard assay described in Methods and % inhibition was calculated in comparison to the untreated enzymatic rate. This resulted in four compounds (**4**, **5**, **6** and **7**) that displayed at least 90% inhibition of enzymatic activity. The other compounds screened displayed 20% or less. Three of the compounds with high inhibitory activity (**4**, **5** and **6**) shared the sulfonamide core. These four compounds were then re-screened with a minimum of six compound concentrations to determine the IC_50_ value (Table 1). The IC_50_ values ranged between 100 μM and 1 mM.

**Table 1:** *In vitro* analysis of small-molecule inhibitors.

Compound No.	HINT	IC_50_ (μM)
Compound **4**	3112	157 ± 26
Compound **5**	3015	310 ± 23
Compound **6**	2876	356 ± 16
Compound **7**	2095	1164 ± 175
Compound **8**	4380	No inhibition at 3 mM
Compound **9**	2570	No inhibition at 3 mM
Compound **10**	4274	2% inhibition at 3 mM
Compound **11**	2344	13% inhibition at 3 mM
Compound **12**	1989	No inhibition at 3 mM
Compound **13**	4190	6% inhibition at 3 mM
Compound **14**	1693	17% inhibition at 3 mM

It is known that compounds identified through large structural databases with high IC_50_ values can be non-specific aggregators, sequestering the enzyme to its surface preventing activity and causing partial denaturation [50, 51]. Aggregation-based inhibition can be reversed through the addition of non-ionic detergents such as Triton X-100. Therefore, to assess the selectivity of our enzyme inhibitor, we re-assayed the dose-dependence with the addition of .01% Triton-X 100, which had no effect on the normal enzymatic activity of *m*-Ddh. The IC_50_ values for Compounds **4**, **5** and **6** showed no difference in activity.

To determine if the small-molecule inhibitors displayed antimicrobial activity, we assessed the minimum inhibitory concentration (MIC) using a standard broth microdilution assay. Compounds **4**, **5** and **6** were tested for their ability to visually inhibit growth of *P*. *gingivalis* cells. Compounds **4** and **5** showed moderate antimicrobial activity with MICs of 250 μM and 167.45 μM, respectively (Table 2). With an MIC over 2 mM, Compound **6** was determined not to be appropriate for whole-cell growth inhibition. These results suggest that the compounds may not be fully permeating the cell membrane or being removed by efflux pumps, preventing the compound from reaching the target. Testing of the minimum bactericidal concentration (MBC) following the MIC assay, showed a MBC to MIC ratio of less than 4 for Compound **4** and **5**. Compound **6** was not screened for bactericidal activity as the concentration higher than the MIC would have been effected by the solvent concentration. The compounds demonstrated select growth inhibition when tested against Gram-positive *S*. *sanguinis* which lacked the target *m*-Ddh. Compound **4** had the greatest difference with 7× MIC of *P*. *gingivalis* and Compound **5** had almost double the MIC (Table 2).

**Table 2:** Minimum Inhibitory Concentration (MIC) and Minimum Bactericidal Concentration (MBC).

	*P*. *gingivalis*		*S*. *sanguinis*
	MIC (μM)	MBC (μM)	MIC (μM)
Compound **4**	250	374	1740
Compound **5**	167	254	305
Compound **6**	2821	**	3310

n = 3

**Effected by solvent concentration

**Fig 6:**
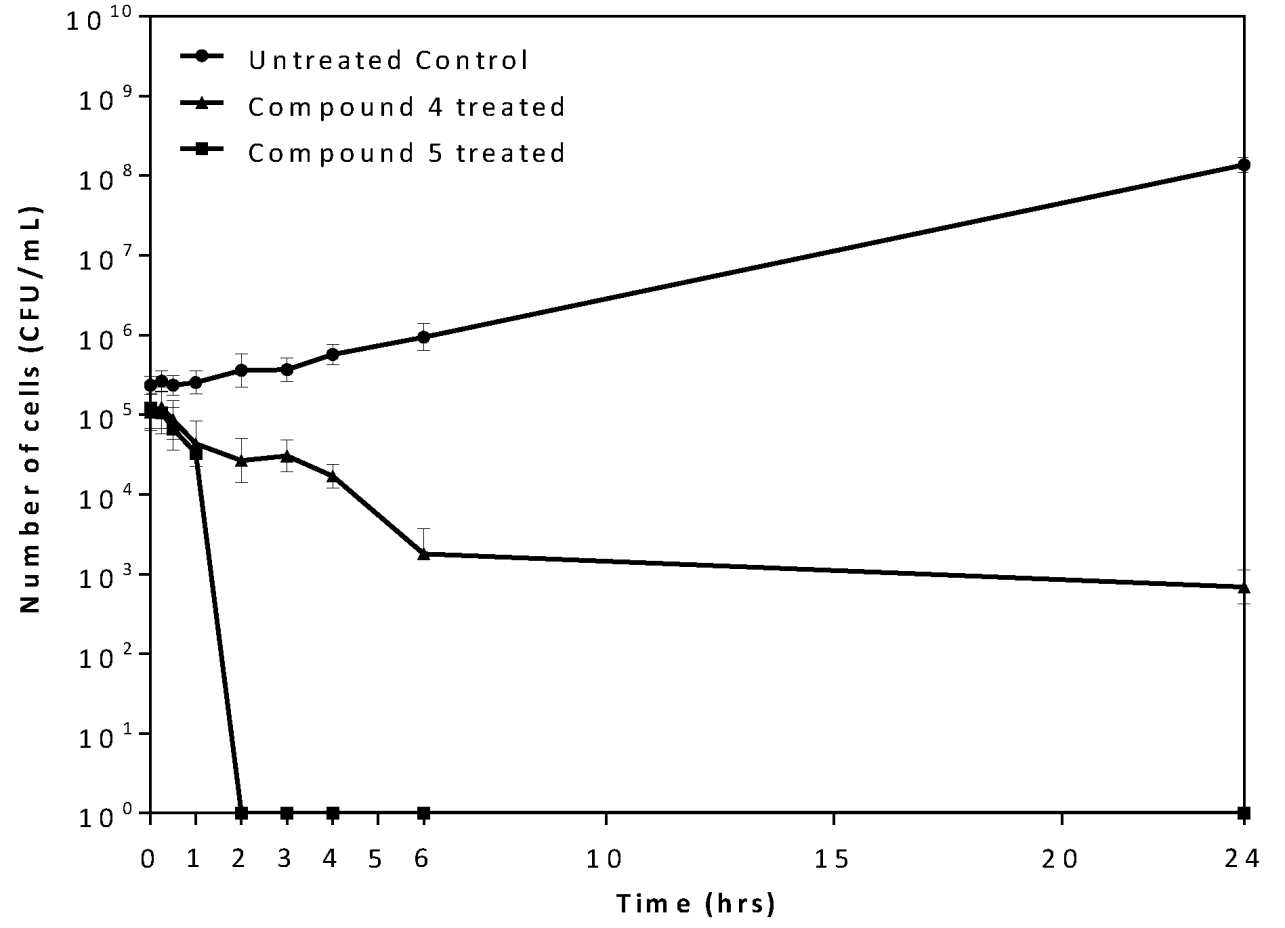
Time-kill analysis of *P*. *gingivalis* treated with Compound 4 and 5. *P*. *gingivalis* cells were treated with 5x the previously determined MIC for either Compound **4** (triangle) or Compound **5** (square) and bacterial cell counts were assessed at 0, 0.25, 0.5, 1, 2, 3, 4, 6 and 24 hours. The mean plus the standard deviation is shown for each time point from a minimum of n = 3 independent experiments. For cell counts equal to 0 CFU/mL, 1 was used for the log transformation.

At 5× MIC, Compound **4** reduced the viable *P*. *gingivalis* cell count by 2 log_10_ CFU /ml within 6 h of exposure. However, Compound **5** rapidly reduced the cell count upon treatment. After 2 h of exposure there was a 5 log_10_ CFU/ml reduction, resulting in no viable cell count (Fig 6). Cells exposed to Compound **6** at the higher concentration treatments were affected by the DMSO solvent and could not be assessed.

**Fig 7:**
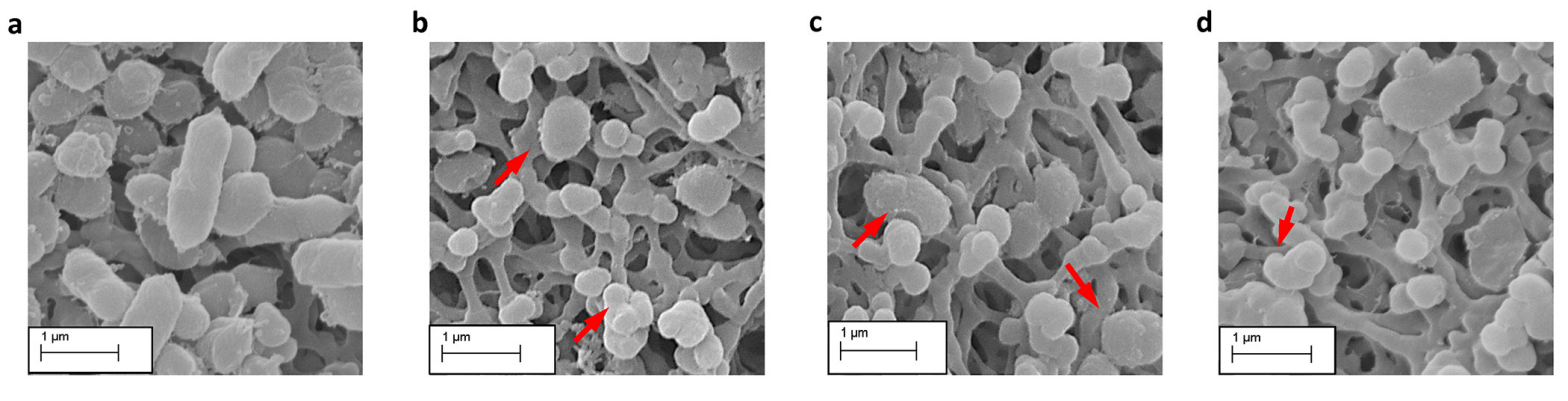
SEM analysis of *P*. *gingivalis* cells treated with Compound 4 and 5. (a) Untreated cells. (b) Compound **4** treated cells at 5x the previously determined MIC concentration. (c) Compound **5** treated cells at 5x the previously determined MIC concentration. (d) Ampicillin treated cells.

Examination of *P*. *gingivalis* cells exposed to either Compound **4** or Compound **5** by scanning electron micrograph (SEM) showed an alteration of the cellular structure (Fig 7). Cells were misshapen with an altered morphology. This was similar to the ampicillin, a cell-wall targeting antibiotic. There were smaller and rounder compared to the wild-type cellular structure seen in the untreated cells.

**Fig 8:**
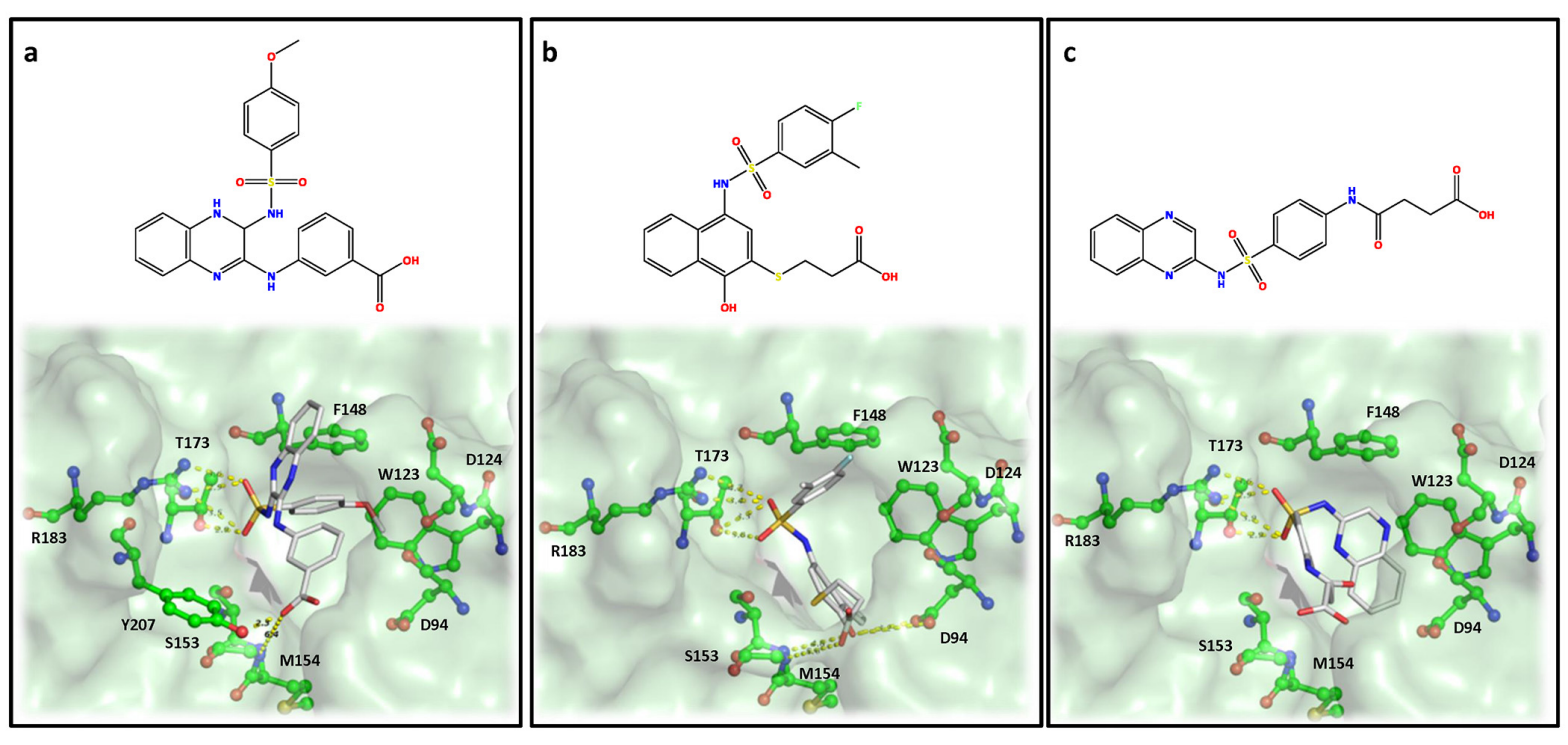
Docking and binding interaction of three active compounds in complex with *m*-Ddh. (a) Compound **4** (b) Compound **5** and (c) Compound **6**. Key residues are labeled, displayed as ball and sticks and colored corresponding to atom type. Hydrogens were omitted for clarity. Potential hydrogen bonding interactions between *m*-Ddh residues and inhibitors are shown by yellow dashed lines.

Analysis of the docking model showed hydrophobic stacking interactions occurring between the aromatic rings of the inhibitors and residues Phe148 and potentially Trp123 of the active site. There was also potential hydrogen bonding between the ligands’ carboxylic groups and residues Gly155 and Met154. The molecular docking and subsequent HINT scoring of Compound **4**, **5** and **6** showed favorable interactions with residues in the *m*-DAP binding site (Fig 8). The sulfonamide group of these compounds showed strong hydrogen-bonding interactions with Arg183 and Thr173, which were shown to be important for *m*-DAP binding. In addition, the carboxylate groups formed hydrogen bonds with the backbone amide of Ser153 and Met154. Also observed were favorable hydrophobic interactions with Trp123 and Phe148. All these interactions followed the proposed pharmacophoric model. Compound **4** made additional hydrogen-bonding interaction with Tyr207 and π-π stacking interactions with Phe148, which may be one of the many reasons for its better activity.

**Fig 9:**
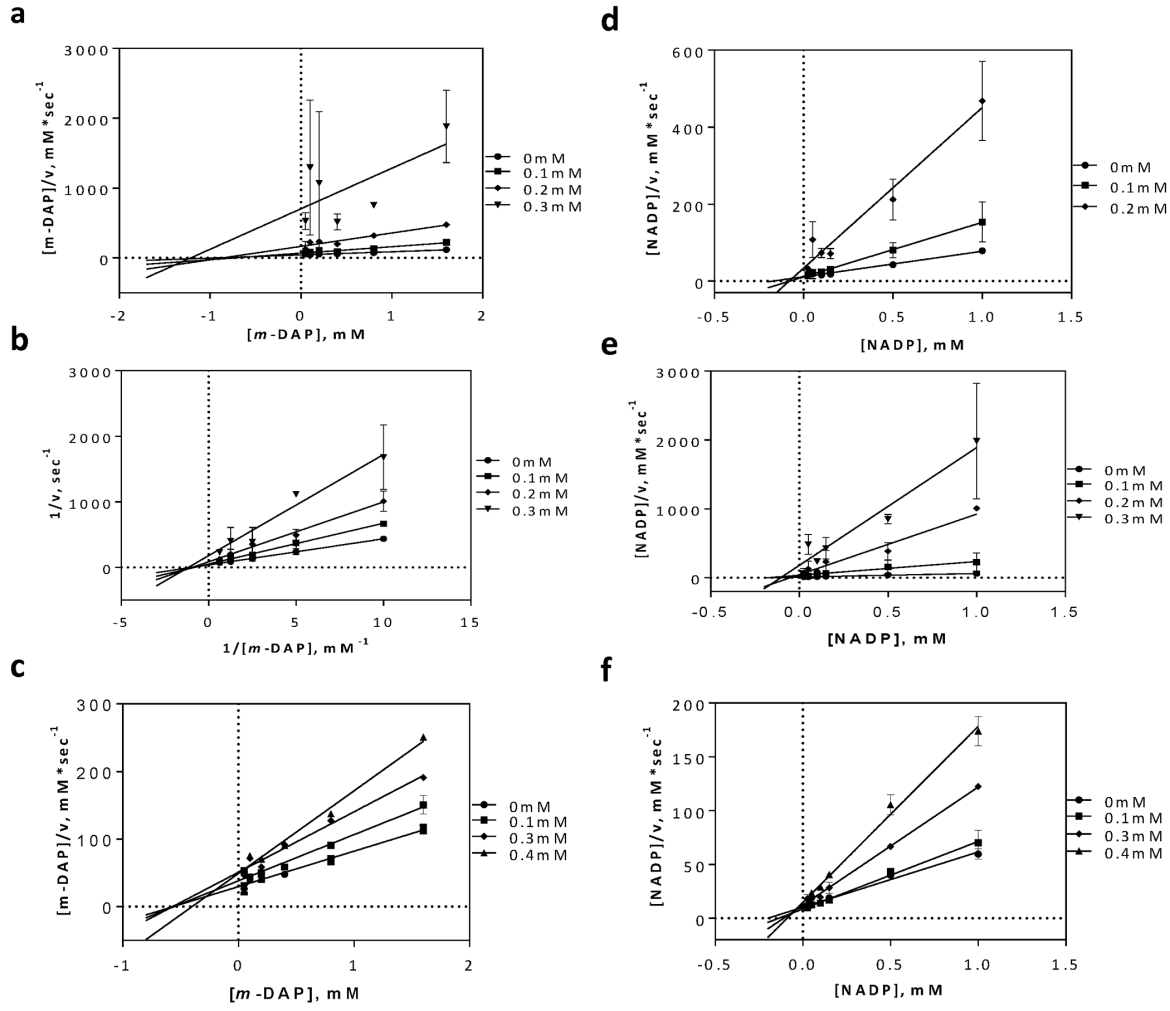
Inhibition mechanism of active compounds in regards to substrate, *m*-DAP and co-substrate, NADP^+^. (a) Compound **4** (b) Compound **5** and (c) Compound **6** inhibition mechanisms against *m*-DAP. (d) Compound **4** (e) Compound **5** and (f) Compound **6** inhibition mechanisms against NADP^+^.

To verify this interaction, inhibition studies were carried out. The substrate *m*-DAP was varied over several concentrations of each inhibitor. The inhibition pattern was revealed to be non-competitive, indicating that none of the compounds were directly competing with *m*-DAP (Fig 9A–9C). This was unexpected with respect to our hypothesis generated from the modeling studies. However, as our inhibitors had moderate activity they may only be weakly competitive against *m*-DAP. Nevertheless, due to structural similarities between the compound scaffolds and the co-substrate, we next examined the possibility that one of more of the inhibitors may be binding in the NADP^+^ binding site. This inhibition study showed an uncompetitive inhibition pattern (Fig 9D–9F).

## Discussion

In this study, we employed a high-throughput virtual screen of more than 9 million small-molecules to identify potential inhibitors against *P*. *gingivalis*. The significance of our study can be summarized as follows: First, we identified *m*-Ddh, an essential enzyme for *P*. *gingivalis*, as a potential pathogen-specific target within the oral cavity. Second, to the best of our knowledge, this is the first computationally motivated target-based drug discovery for this periodontal pathogen. Third, we show that we can identify through computational means important pharmacophore features for substrate-protein interactions and identify inhibitors with drug-like properties that can be further optimized for select *in vitro* activity. Finally, *P*. *gingivalis* and periodontal disease can be used as the starting model for rational species-selective drug discovery, combing genomic knowledge of essential genes and computational advances for small-molecule identification.

We hypothesized that we could selectively target periodontal pathogens in the oral cavity by excluding genes essential for non-pathogenic early oral colonizers. Essential genes from *S*. *sanguinis* [14] represented a healthy oral cavity and we compared potential essential gene targets from *P*. *gingivalis*, to generate a subset of genes predicted to be essential in the periodontal pathogen while potentially absent within a healthy cavity. We then identified *m*-Ddh as a potential pathogen-specific target for the control of periodontitis and we believe it represents a unique and promising target. First, it is important for bacteria as *m*-Ddh is found within the lysine biosynthesis pathway and catalyzes the reversible NADP^+^-dependent oxidative deamination of *m*-DAP. *m*-DAP is vital as it is a direct precursor to the amino acid L-lysine and a key component in peptidoglycan biosynthesis by cross-linking the glycan backbone in the cell wall of mycobacteria and Gram-negative bacteria, leading to the cellular strength and structure [39, 52]. Especially interesting is the dual role *m*-Ddh plays in lysine and peptidoglycan biosynthesis, making evident its potential for antimicrobial therapy. Second, it is only known to be present within a small fraction of bacterial species, mostly limited to only a few *Bacillus* species [40, 53]. Nevertheless, in the context of periodontal disease, *m*-Ddh has potential for a broader application. A BLASTP of completed oral genomes in the Human Oral Microbiome Database (HOMD) [54] showed out of 315 sequenced genomes, 69 possessed a highly conserved sequence for *m*-Ddh. Many of those observed were other known oral pathogens such as *Prevotella* sp., *Tannerella* sp. and *Veillonella* species. Third, it presents no human homologue. The biosynthetic pathway for lysine is completely lacking in mammals, indicating a lower chance of toxicity. Fourth, the idea of disrupting lysine biosynthesis as a site for antimicrobial and antifungal targets is not new. Numerous studies have been published analyzing enzymes within lysine pathways as potential targets [39, 53, 55, 56]. Lysine riboswitches were identified as potential targets in *Bacillus*, where several lysine analogs were shown to inhibit bacterial growth *in vitro* [57]. Studies have also been pursued for antifungals. Deletion of homocitrate synthase, an essential enzyme in the aminoadipate pathway for *Aspergillus fumigatus*, showed reduced virulence in a bronchopulmonary aspergillosis mouse model [56]. Lastly, it possesses key features corresponding to that of a protein target.

When selecting a drug target, it must one, be essential for the survival of the pathogen or disease virulence; two, possess certain sequence and structural features and three, have assayable activity. We first confirmed the essential nature of *m*-Ddh through allelic replacement mutagenesis. This method allowed us to rapidly evaluate the effect of a single gene knock-out in *P*. *gingivalis* while minimizing possible polar effects. Additionally, the essential nature of a gene can be determined with a distinct phenotype corresponding to colony growth or lack thereof. We observed no growth for *m*-Ddh. This was compared to a hypothetical membrane protein that was not essential for *P*. *gingivalis* survival [58, 59]. We previously used this method to systematically knock-out over 2000 genes in *S*. *sanguinis* for the genome-wide identification of essential genes [14]. It has also been applied to several other essential gene studies, including *E*. *coli*, *B*. *subtilis* and *S*. *pneumoniae* [60–62]. Previous studies comparing sequence and structural data between targets and non-targets showed druggable proteins are more likely to be of certain enzyme classes, contain more non-polar amino acids and have a lower pI, indicating molecules more acidic in nature [46]. Based on these factors, *m*-Ddh is a “druggable” enzyme with a sequence and structural motif that has the ability to be targeted by small-molecules. Previous studies of *m*-Ddh and its role in lysine biosynthesis have focused on the enzyme from *Corynebacterium* [31], *Bacillus* [41], and *Ureibacillus* [52]. However, there is no data on *m*-Ddh in *P*. *gingivialis* and while the crystal structure was published in PDB, prior to our study, data concerning the physiochemical and kinetic properties of *m*-Ddh for *P*. *gingivalis* was unknown. The apparent K_m_ appears to vary between *m*-Ddh of different species; however, the values for *P*. *gingivalis* were consistent with *C*. *thermocellum* which was reported K_m_ values of 230 μM and 90 μM for NADP^+^ [40].

Historically, traditional antimicrobial drug studies focused on screening large numbers of compounds for whole-cell activity, flushing out the mechanism of action and verifying the feasibility of the target later. With the crystal structure available for our target, we decided to utilize a structure-based drug design approach. A subset of compounds with the most favorable docking interactions determined through HINT interaction scores were selected for our initial screening. From 11 compounds identified and screened *in vitro*, compound **4**, **5**, **6** and **7** showed target-specific inhibition. Three of the four (**4**, **5** and **6**) displayed IC_50_ values in the mid micromolar range (Table 1). This high hit rate of around 30%, is impressive for an initial screen. While the HINT and docking analysis did predict several molecules to have better interactions, these did not have *in vitro* activity. However, HINT assess binding affinity which does not always correlate with activity. A re-evaluation of the virtual screening query could significantly lead to more compound hits. The compounds with inhibitory activity showed limited structural similarity, but possessed similar functional groups and were predicted to share basic pharmacophore features. They all possessed sulfonamide core attached to large aromatic structures with carboxylate functional groups. The importance of the sulfonamide has precedence in the search for antimicrobials targeting lysine biosynthesis. Compounds structurally similar to our hits that possessed sulfonamides and sulfones were identified as fairly good inhibitors of dihydrodipicolinate reductase, another enzyme in the lysine biosynthesis pathway [53, 63]. Based on docking studies, the sulfonamide groups were predicted to favorably interact with Arg183 and Thr173 forming hydrogen bonds. The aromatic moieties would create hydrophobic interactions and the carboxylic groups would form hydrogen bonds with residues at the other end of the binding pocket. It should be noted that previous studies have reported more potent inhibitors against *m*-Ddh [47, 53, 64]. However, these compounds are typically small analogous structures, derived from the substrate *m*-DAP that possess few ‘drug-like’ features, making optimization difficult [53] and suggesting little hope for selectivity. Our compounds allow for the development of more active compounds. This is similar to the *in silico* screening against thymidylate synthase, an enzyme is essential for DNA replication, by DesJarlais *et al*. [65]. The initial computational study yielded several compounds with activity in the high micromolar range, but following further analysis and optimization resulted in an increase in potency as well as verification of the binding mode.

Previous initial-velocity data [28] in *m*-Ddh show the reaction to proceed through a sequential ordered ternary-binary mechanism with NADP^+^ binding first, followed by the substrate *m*-DAP. The product is then released, followed by NADPH. Our studies into the mechanism of inhibition (Fig 9) showed the molecules to be non-competitive with respect to *m*-DAP, but uncompetitive with respect to NADP^+^. In concordance with the binding order, this would indicate that the inhibitors bind to either the Enzyme-NADP^+^ complex or the Enzyme-NADPH complex, thus potentially preventing a necessary conformational change and/or reducing the affinity of *m*-DAP for the protein. This type of mechanism of inhibition could be beneficial for future therapeutics. Treatment with an optimized inhibitor competing with *m*-DAP, would result in the accumulation of the substrate within the cytosol. An increase in the localized substrate would then need to be balance by high concentrations of the inhibitor. A non-competitive inhibitor, however would not be affected by the increased concentration of substrate compared to a competitive inhibitor, making it more effective at lower concentrations.

One of the most difficult aspects of target-based drug discovery is identifying small-molecules that show effective whole-cell activity while maintaining the key pharmacokinetics. While the inhibitors identified showed potential against *m*-Ddh, they exhibited moderate antimicrobial activity in *P*. *gingivalis* (Table 2). Nevertheless, the potential for antimicrobial activity should not ignored as analogous structures and optimization of the scaffold could improve whole cell inhibition. Several reasons could contribute to the high MIC values. For one, bacterial inhibitors must be able to penetrate the cell membrane while maintaining enough soluble and free fractions to inhibit the target at sufficient concentrations. The compound also must avoid being expelled from the cell through efflux pumps. Another reason could be due to the sulfonamide group present on compound **4**, **5** and **6**. Sulfonamide derivatives are well known antimicrobials that target folate biosynthesis, and bacterial cells may show a degree of drug resistance similar to others studies in lysine inhibition [53]. There is also the potential for non-specific inhibition or off-target interactions. This would result in what appears to be a change in activity during whole-cell inhibition compared to the target inhibition. This was observed for compound **5** which displayed a lower MIC than the IC_50_. While a detailed structure-activity relationship for the antimicrobial properties cannot be determined from these studies, it may be speculated that the more favorable whole-cell activity seen in Compound **5** compared to compound **4** and **6** is due to lipophilicity. The relatively low lipophilic nature of compound **6** (cLogP = 1.70) compared to **5** (cLogP = 3.68) may have decreased permeability through the cellular membrane of *P*. *gingivalis*. While compound **4** displayed the more potent target-based screening, the high lipophilic nature (cLogP = 5.26) may have had a significant effect on the solubility, reducing the efficacy during cell-based screening [66].

We were able to show differential activity indicating potential specificity. Testing in *S*. *sanguinis*, which lacks the target *m*-Ddh, showed almost no antimicrobial activity with MICs more than double that of those seen in *P*. *gingivalis*. For Compound **5**, complete elimination of *P*. *gingivalis* cell viability was achieved after two hours of exposure at 5x the MIC concentration, while Compound **4** maintained a low cell count after six hours of treatment. Therefore, the activity of these compounds is time and dose dependent, with higher concentration and longer exposure times leading to an increase loss of cell viability. This would indicate that these compounds would most likely be killing the cell at working concentrations in agreement with other cell well targeting antibiotics. This corresponds with the MBC being less than 4x the MIC as antimicrobials with MBC in close range of the MIC are typically classified as bactericidal. The changes observed in the cell wall morphology for the compound treated *P*. *gingivalis* cells corresponded to targeting of the cell wall as well as disruption in protein synthesis. Similar cell wall alterations were in seen in *E*. *coli* when treated with ribosome targeting antibiotics [67]. This would be consistent with *m*-Ddh inhibition, as it plays a dual role in peptidoglycan and lysine biosynthesis.

In conclusion, our results demonstrate the possibility of identifying inhibitors that can target *P*. *gingivalis m*-Ddh. We show that these inhibitors bind *m*-Ddh and prevent the enzymatic reaction from occurring. Continued studies into the protein-inhibitor binding interaction could help to discern which features are key, allowing for the development of improved inhibitors. The current overuse and misuse of broad-spectrum antibiotics has led to a steady increase in bacterial resistance and contributes to adverse health effects such as gastrointestinal infections [7, 8]. Yet, there has been a surprising lack of antimicrobial drug development especially among Gram-negative bacteria, which have seen rises in multi- and pan-drug resistant strains. Pathogen-specific antimicrobial therapy could help generate novel leads in drug discovery, ease resistance commonly seen in broad-spectrum antibiotics and reduce the time for the discovery as targets won’t have to be verified across multiple species. Since *m*-Ddh is a unique target found within a limited number of species, we believe that our current effort could serve as a proof-of-concept and lead to the development of novel narrow-spectrum therapy for the treatment of periodontal disease.

## Supporting Information

S1 Fig SDS PAGE analysis of purified protein. M1, Precision Plus Protein Dual Color Markers (Bio-Rad); M2, Precision Plus Protein Dual Xtra Prestained Protein Markers (Bio-Rad); TCP, total cell protein; S, soluble fraction; FT, flow-thru; E, empty lane; P, purified protein via His-tag. Arrow represents target protein.
(TIF)

S2 Fig Dose-dependent analysis of unsaturated analogs of *m*-DAP against *P*. *gingivalis m*-Ddh. (TIF)

S3 Fig Characterization of kinetic properties of *P*. *gingivalis m*-Ddh. (TIF)
