# Supplementary figures and images for: Identification of Small-Molecule Inhibitors against Meso-2, 6-Diaminopimelate Dehydrogenase from Porphyromonas gingivalis
Source: PLoS One. 2015 Nov 6;10(11):e0141126. doi: 10.1371/journal.pone.0141126 (PMC4636305; doi:10.1371/journal.pone.0141126)

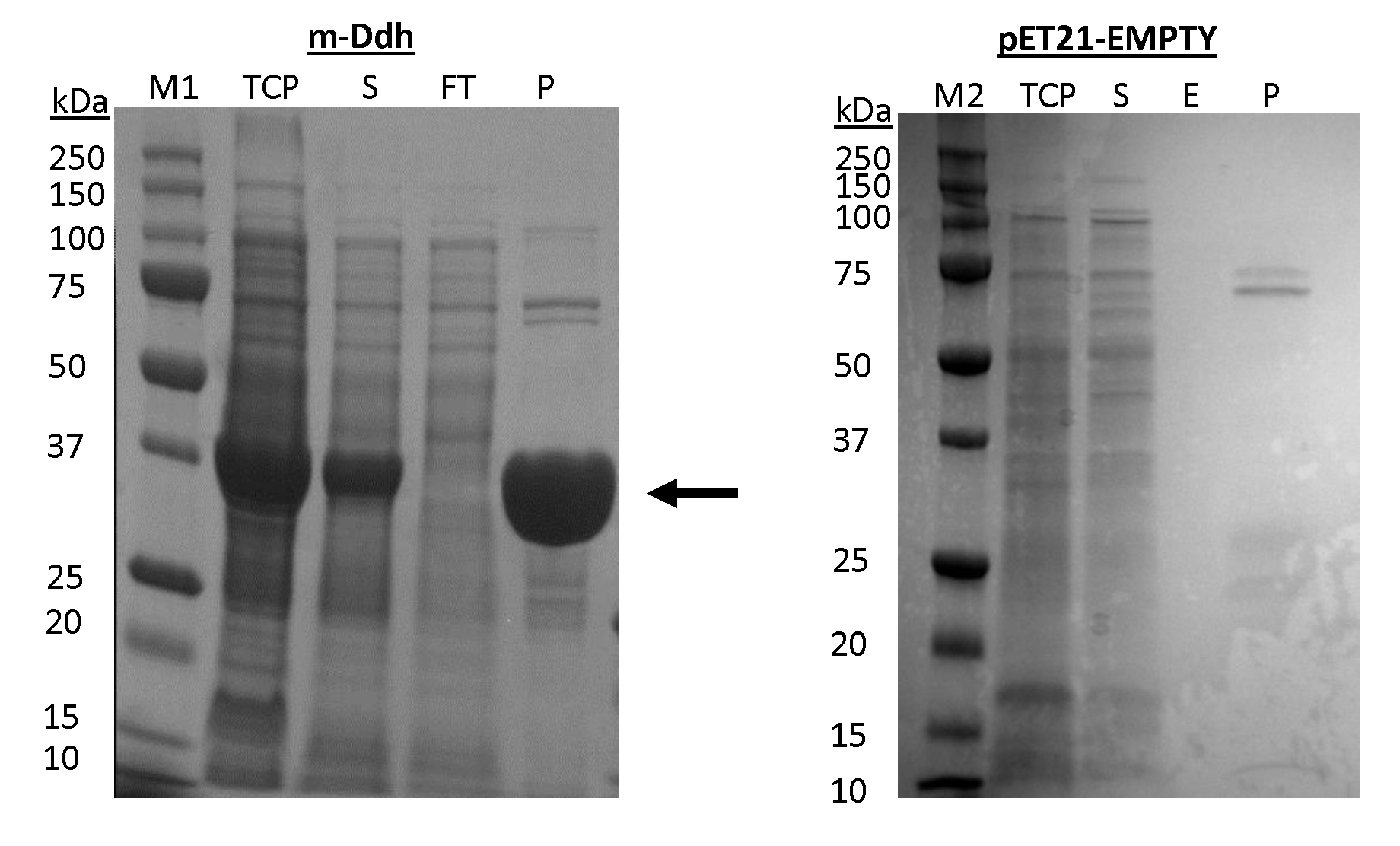

Supplement: S1 Fig — M1, Precision Plus Protein Dual Color Markers (Bio-Rad); M2, Precision Plus Protein Dual Xtra Prestained Protein Markers (Bio-Rad); TCP, total cell protein; S, soluble fraction; FT, flow-thru; E, empty lane; P, purified protein via His-tag. Arrow represents target protein. (TIF) [file pone.0141126.s001.tif]

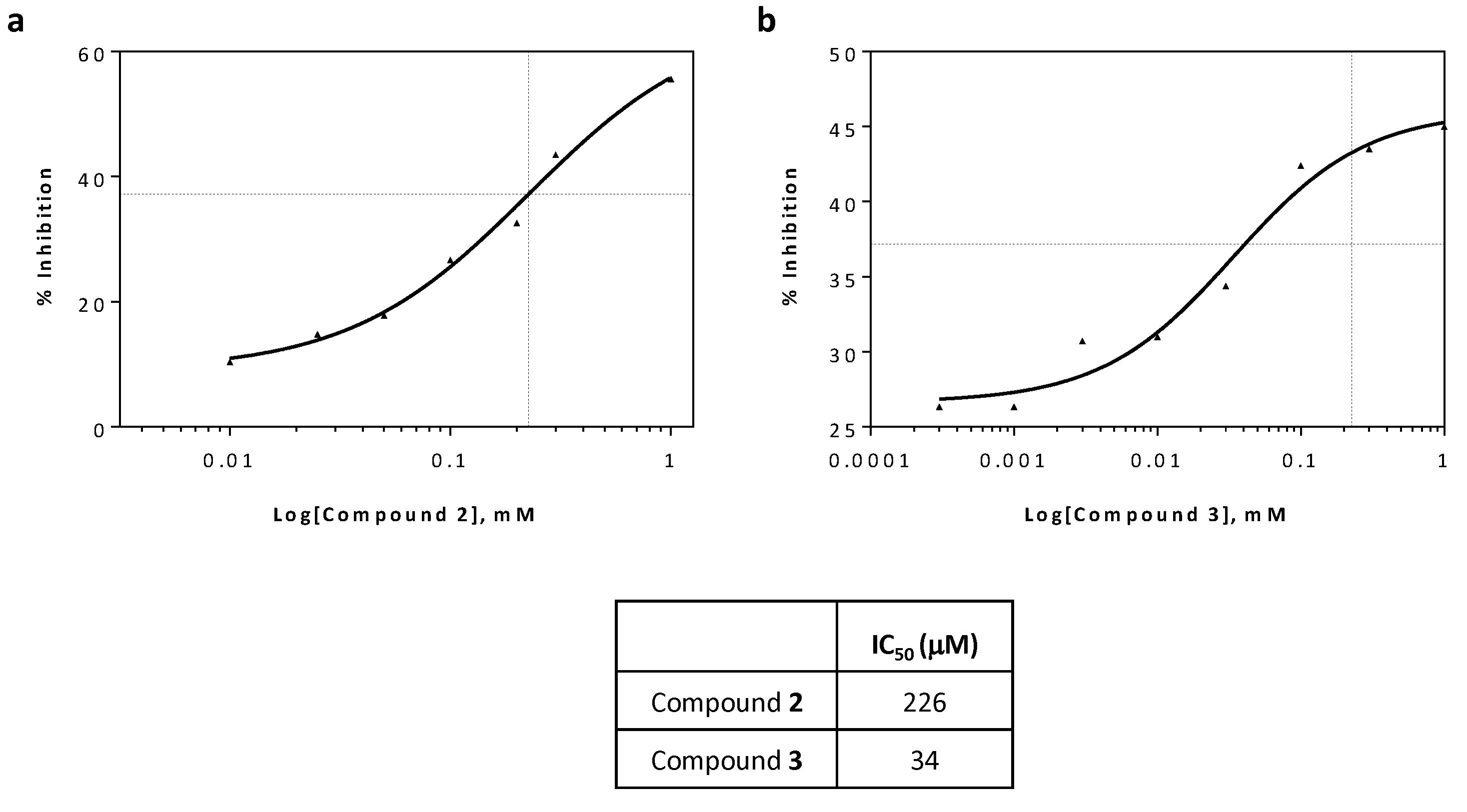

Supplement: S2 Fig — (TIF) [file pone.0141126.s002.tif]

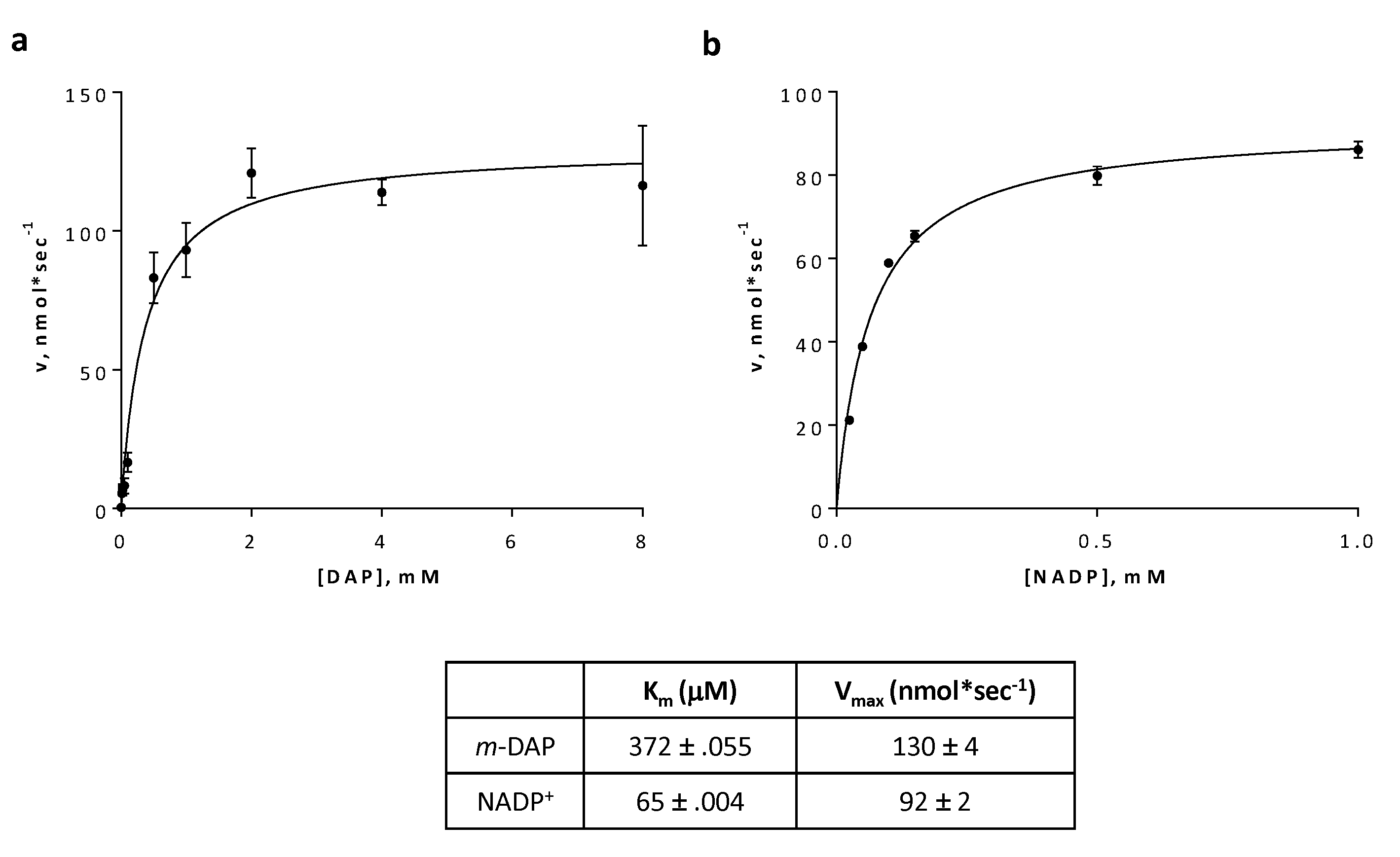

Supplement: S3 Fig — (TIF) [file pone.0141126.s003.tif]
